# Effect of microencapsulated essential oil form *Chamaecyparis obtusa* on monocyte-derived dendritic cell activation and CD4^+^ T cell polarization

**DOI:** 10.1371/journal.pone.0201233

**Published:** 2018-07-27

**Authors:** Seung-Heon Shin, Mi-Kyung Ye, Dong-Won Lee, Mi-Hyun Che

**Affiliations:** Department of Otolaryngology-Head and Neck Surgery, School of Medicine, Catholic University of Daegu, Daegu, South Korea; Auburn University College of Veterinary Medicine, UNITED STATES

## Abstract

The essential oil of *Chamaecyparis obtusa* (*C*. *obtusa*), which is used in soap, toothpaste, and aromatic agents, has been known to have anti-inflammatory properties. In this study, we investigated the effects of microencapsulated *C*. *obtusa* essential oil on airborne fungus-induced dendritic cell (DC) activation and Th immune responses. We stimulated monocyte-derived DCs with *Alternaria* alternate and lipopolysaccharide (LPS). To determine the anti-inflammatory effects, we pre-treated DCs with various concentrations of microencapsulated *C*. *obtusa* essential oil and collected the supernatants to measure interleukin-6 (IL-6) and tumor necrosis factor-alpha (TNF-α), and we determined the expression of cell surface molecules. The effects of the essential oil on CD4^+^ T cells polarization was determine by culturing stimulated DCs and autologous CD4^+^ T cells. *Alternaria* enhanced the production of IL-6 and TNF-α from DCs, and pretreating DCs with 0.001, 0.01, and 0.05% of the essential oil significantly inhibited their production. Increased CD80 and CD86 expression by *Alternaria* was significantly inhibited with 0.05% of the essential oil. *Alternaria*-induced IL-5, IL-10, and interferon-gamma from CD4^+^ T cells were significantly inhibited with *C*. *obtusa* essential oil in a dose dependent manner. *C*. *obtusa* influenced both *Alternaria*- and LPS-induced Th1 and Th2 polarization of CD4^+^ T cells. These results suggest a novel pharmacological use for *C*. *obtusa* essential oil to treat inflammatory airway diseases.

## Introduction

A number of environmental factors are associated with allergic airway disease. House dust mites and mold are significant airborne allergens for developing allergic rhinitis and asthma. Most fungi are ubiquitous in the environment, and they enter the respiratory tract by means of inhalation. Among several pathogenic fungi, *Alternaria* and *Aspergillus* are commonly found in the airway and trigger airway diseases. [1, 2] Severe life-threating asthma and eosinophilic airway inflammation have been associated increased airborne exposure to *Alternaria*. [3, 4] Fungi contain several immunologic factors that are potent regulators of the host immune response. These immunologic factors, with pathogen-associated molecular patterns, interact with pattern recognition receptors in antigen-presenting cells. [5] Multiple fungal signaling pathways in these cells affect the local Th cell balance. For example, inflammatory dendritic cells (DCs) initiate antifungal response in the Th17 and Th2 signaling pathways, and tolerogenic DCs activate Th1 and Treg cell immune response. [2] *Alternaria* can develop Th2-type immune response in the airway by activating dendritic cells. [1]

Phytotherapy is alternative medicine that uses extracts from natural products that are known to promote health with varying benefits and lack of side effects. [6] Essential oils from trees and plants have protective mechanisms against harmful insects and microorganisms, and a number of essential oils have shown anti-bacterial, anti-fungal, anti-oxidative, and anti-inflammatory activity. [7–9] *Chamaecyparis obtusa* (*C*. *obtusa*) is a tropical tree species found in the southern part of South Korea. The essential oil of *C*. *obtusa* contains several monoterpene hydrocarbons, oxygenated monoterpenes, sesquiterpene hydrocarbons, oxygenated sesquiterpenes, and others. [10, 11] The essential oil of *C*. *obtusa* has been applied in soap, toothpaste, and aromatic agents with anti-oxidative and anti-inflammatory effects. [9, 12] *C*. *obtusa* has been demonstrated to reduce the production of prostaglandin (PG) E2, tumor necrosis factor-α (TNF-α), interleukine-6 (IL-6), and cyclooxygenase-2 (COX-2) in peripheral blood mononuclear cells in a murine model of inflammation. [12, 13]

Although the biological activities of C. obtuse essential oil are not fully understand, antimicrobial, anti-fungal, and anti-nociceptive effects have been studied. [9, 10] However, the anti-inflammatory or immune modulatory effect of C. obtuse essential oil on airway mucosa is not commonly studied. In the present study, we investigated the anti-inflammatory effects and the influence on Th cell polarization of *C*. *obtusa* essential oil in airborne fungi and Gram-negative bacteria induced monocyte-derived DC activation and Th immune responses by determining the expression of inflammatory chemical mediators and Th cytokines.

## Materials and methods

### Preparation of the C. *obtusa* essential oil

Qwell Inc. (Seoul, South Korea) kindly presented Microencapsulated *C*. *obtusa* essential oil; it was isolated from leaves collected in Masan, Kyunsangnamdo, South Korea. The leaves dried under shade, and using the seam distillation and extraction methods, they extracted essential oil from the leaves. The collected essential oil was stored at room temperature for one year to stabilize its components. The chemical composition of *C*. *obtusa* essential oil was determined using a gas chromatography mass spectrometry analysis (Agilent Tech., Santa Clara, CA, USA). Essential oil contained more than 20 components and the main chemical composition of *C*. *obtusa* essential oil is in Table 1. For microencapsulation, they emulsified the oil by mixing it with a water-soluble stylene maleic anhydride polymer in water and then adding melanin pre-condensate to the mixture, resulting in the formation of microcapsules consisting of a spherical inner core and an outer shell surrounding the inner core.

**Table 1:** Main components of essential oil from *Chamaecyparis obtusa*.

Compound	Retention time (min)	Peak area (%)
α-Pinene	8.75	5.96
Sabinene	10.96	16.72
Myrcene	11.36	19.45
α-Terpinene	12.69	3.05
γ-Terpinene	13.54	4.75
Limonene	13.76	1.82
Terepineol	15.21	1.26
Terpinene-4-ol	16.23	2.82
Bornyl acetate	27.53	9.46
α -Terpinyl acetate	28.64	15.69

### Generation of the monocyte-derived DCs

We isolated peripheral blood mononuclear cells (PBMCs) from normal healthy volunteers after they provided informed consent. The procedure was approved by the Institutional Review Board of Daegu Catholic University Medical Center. A duly completed written informed consent form that outlined the objectives of the research and experiments was obtained from each subject. The cells were isolated by density gradient centrifugation on Histopaque (Sigma-Aldrich, St. Louis, MO, USA). We harvested cells from the interphase and washed them with phosphate-buffered solution, and then we mixed the PBMCs with anti-CD14 antibody conjugated with magnetic particles (Miltenyl Biotec, Sunnyvale, CA, USA) at 4°C for 30 minutes to obtain monocytes. We cultured the positively collected cells with 25 ng/ml of granulocyte-macrophage colony-stimulating factor and 10 ng/ml of interleukin-4 (IL-4; R&D Systems, Minneapolis, MN, USA). After 6 days of culture, we harvested the DC-enriched portion and analyzed as previously reported. [14] Cells differentiation and purity were determined with flow cytometry (Becton Dickinson, Mountain View, CA, USA). We used cells preparation contained more than 90% of DC phenotype CD11c.

### Cyotoxicity of *C*. *obtusa* essential oil

We determined the cellular cytotoxicity of the microencapsulated essential oil with monocyte-derived DCs and CD4^+^ T cells. Cells were plated into the wells of 96-well tissue culture plates (DCs at 1 × 10^6^/mL, CD4^+^ T cells at 2 × 10^6^/mL,) and cultured in the presence of 0.05 to 1% of essential oil (DCs for 24 hours, CD4^+^ T cells for 72 hours) at 37°C in a humidified 5% CO2 chamber. We measured cytotoxicity using a CellTiter-96^®^ aqueous cell proliferation assay kit (Promega, Madison, WI, USA). The tetrazolium compound and Owen’s reagent were added to each well and incubated the plate for 4 hours, and the color intensities were assessed with a microplate reader at 490 nm wavelength (Molecular Devices, Sunnyvale, CA, USA).

### *C*. *obtusa* essential oil inhibition of DC activation

To investigate the anti-inflammatory effects of *C*. *obtusa* essential oil, we suspended 1 × 10^6^/mL of the monocyte-derived DCs in RPMI-1640 with 10% fetal bovine serum and pretreated them with various concentrations of the essential oil for 1 hour and then stimulated the mixture with 50 ug/ml of endotoxin-removed *Alternaria alternata* (Greer Lab., Lenoir, NC, USA) or 1 ug/ml of lipopolysaccharide (LPS; Sigma-Aldrich) for 6 hours.

We assessed the effect of *C*. *obtusa* essential oil on the production of cytokines in DCs to *Alternaria* and LPS stimulation with ELISA and the expression of cell surface molecules with fluorescence-activated cell sorting (FACS). For cytokine analysis, we analyzed cell-free supernatants for IL-6 and TNF-α with a commercially available ELISA assay kit (R&D Systems). For FACS analysis, the DCs were stained with PE-conjugated anti-CD11c and FITC-conjugated anti-CD 80, anti-CD 86, and anti-HLA-DR and with corresponding isotype-matched control monoclonal antibodies. We obtained all antibodies used for FACS analysis from Becton Dickinson and conducted the analysis with a FACScan flow cytometer (Becton Dickinson). We expressed the mean fluorescence intensities of costimulatory molecules as a percentage of treated versus non-treated cells.

### Effects of *C*. *obtusa* essential oil on DCs and CD4^+^ T cell coculture

We measured the effects of the essential oil on CD4^+^ T cells polarization based on cytokine production by culturing monocyte-derived DCs and autologous CD4^+^ T cells. We isolated the CD4^+^ T cells by treating the PBMCs with anti-CD4 antibody conjugated with magnetic particles (Miltenyl Biotec). We cultured CD4^+^ T cells at a ratio of 10:1 with stimulated DCs pulsed with *Alternaria* or LPS for 72 hours. We measured the cytokine production in the cell-free supernatant for IL-5, IL-10, and interferon-gamma (INF-γ; R&D systems).

### Statistics

The results are expressed as mean ± standard error of mean and reflect six independent experiments. We performed one-way analysis of variance followed by Tukey’s test for normally distributed data and the Kruskal-Wallis tests with post-hoc Bonferroni-Dunn testing for non-normally distributed data (SPSS Inc., Chicago, IL, USA) and considered *p* less than 0.05 statistically significant.

## Results

### Cytotoxicity of *C*. *obtusa* essential oil

**Fig 1:**
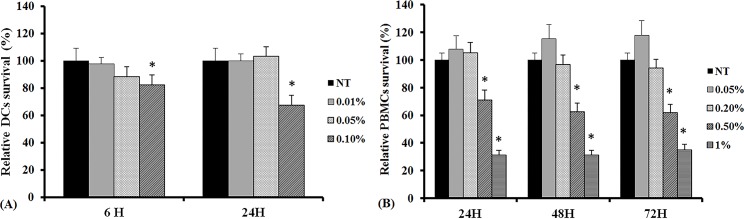
Effects of *C*. *obtusa* essential oil on survival of dendritic cells (DCs) and peripheral blood mononuclear cells (PBMCs). DC survival decreased significantly with 0.1% of the essential oil (A), and PBMC survival decreased significantly at concentration exceeding 0.5% (B). Exposure time did not influence cell survival. NT: not treated with essential oil, H: hours, * p<0.05.

In order to determine the optimal concentration of essential oil, we treated DCs and the CD4^+^ T cells for 24 and 72 hours with different concentration of the essential oil. The DCs’ viability decreased significantly with an oil concentration of 0.1% (82.3 ± 7.9 after 6 hours and 67.3 ± 6.4 after 24 hours). The exposure time did not influence the DCs’ survival. When the CD4^+^ T cells were treated with 0.05, 0.2, 0.5, and 1% of essential oil for 24, 48, and 72 hours, CD4^+^ T cells survival decreased significantly with 0.5% and 1% of the oil, and again the exposure time did not influence cell viability (Fig 1).

### Inhibition of DCs activation with *C*. *obtusa* essential oil

**Fig 2:**
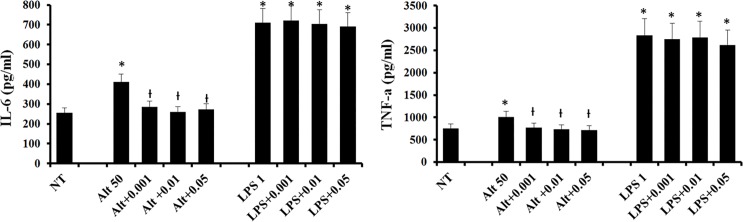
Effects of *C*. *obtusa* essential oil on chemical mediator production from dendritic cells (DCs). When DCs were pretreated with various concentrations (0.001–0.05%) of *C*. *obtusa*, interleukin-6 (IL-6) and tumor necrosis factor-α (TNF-α) induced by *Alternaria* 50 ug/ml (Alt 50) was significantly inhibited. However, chemical mediator production induced by lipopolysaccharide 1 ug/ml (LPS 1) was not inhibited. NT: not treated with essential oil, * p < 0.05 vs. NC, Ɨ: p < 0.05 vs. Alt 50 or LPS 1.

We pretreated the DCs with the essential oil for 1 hour, and then stimulated them with *Alternaria* or LPS for 6 hours. DCs had been reported to produce IL-6 and TNF-α, and these cytokines play important roles in regulating T cell differentiation and inflammation. *Alternaria* and LPS significantly increased the production of IL-6 (*Alternaria*: 410.9 ± 52.7 pg/ml, LPS: 711.6 ± 122.3 pg/ml) and TNF-α (*Alternaria*: 1007.2 ± 253.7 pg/ml, LPS: 2837.6 ± 357.2 pg/ml) compared with the untreated group (IL-6: 255.6 ± 43.6 pg/ml, TNF-α: 752.6 ± 176.6 pg/ml; p < 0.05). When the DCs were pretreated with 0.001, 0.01, and 0.05% of the oil, *Alternaria* induced IL-6 and TNF-α productions were significantly inhibited. However, the *C*. *obtusa* essential oil did not suppress either LPS-induced IL-6 or TNF-α productions (Fig 2).

**Fig 3:**
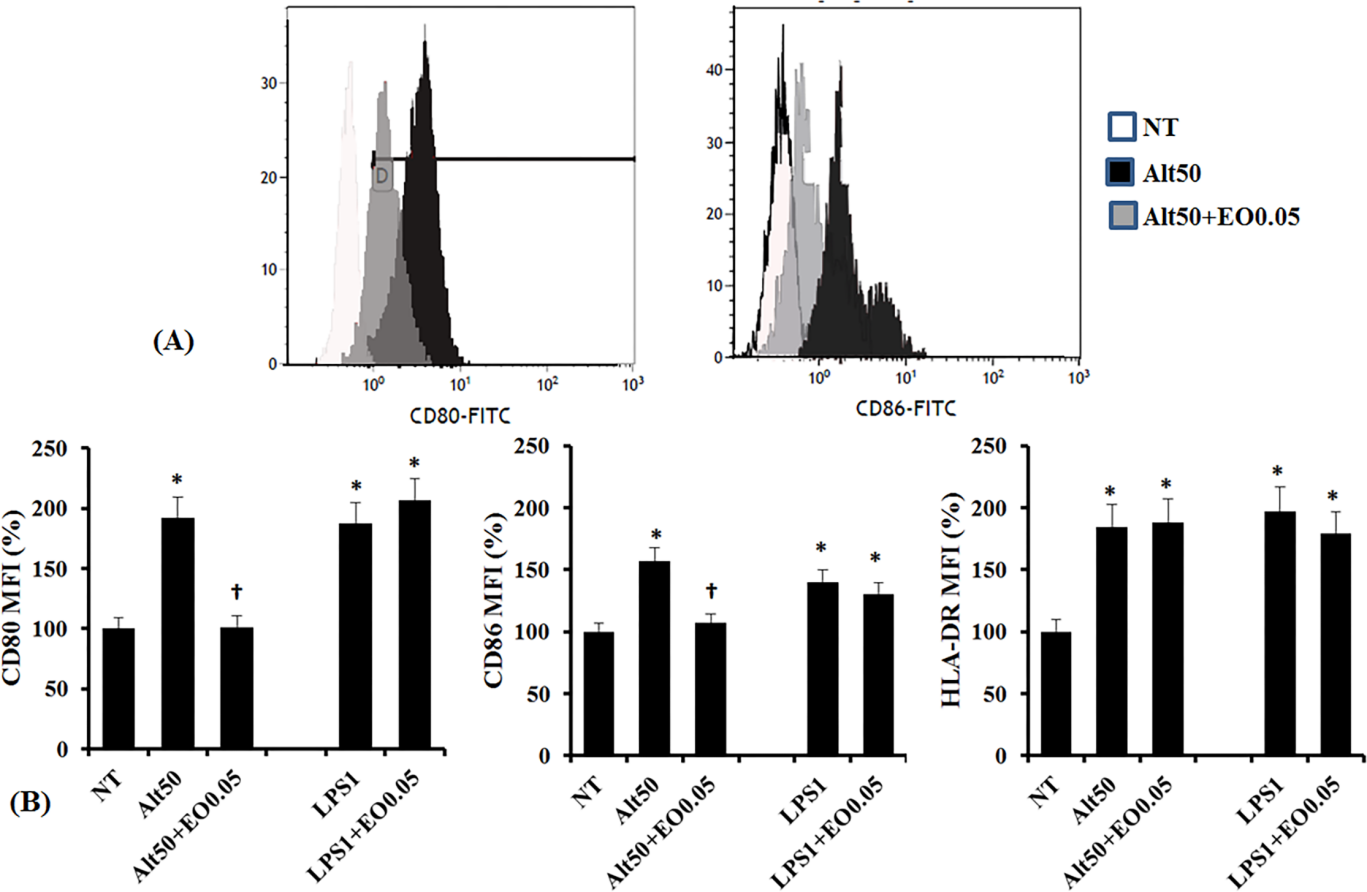
Effects of *C*. *obtusa* essential oil on expression of co-stimulatory molecules by dendritic cells (DCs) and chemical mediator production from DCs. *Alternaria* 50 ug/ml (Alt 50) and lipopolysaccharide 1 ug/ml (LPS 1) significantly enhanced CD80, CD86, and HLA-DR expression, but 0.05% of the essential oil (EO0.05) only inhibited the expression of CD80 and CD86. (A) Representative flow cytometric histograms show expression of costimulatory molecules incubated with *Alternaria* alone or with EO0.005 pretreatment. (B) Mean fluorescence intensity (MFI) levels are shown from five independent experiments. NT: not treated with essential oil, * p < 0.05 vs. NC, Ɨ: p < 0.05 vs. Alt 50 or LPS 1.

When the DCs were incubated for 6 hours with *Alternaria*, the expression of costimulatory molecules including CD80, CD86, and HLA-DR was increased. LPS also increased the expression of CD80 and HLA-DR but not CD86 in DCs. Increased CD80 and CD86 expression by *Alternaria* was significantly inhibited with 0.05% of the essential oil, but the oil did not influence HLA-DR expression in the DCs. The *C*. *obtusa* essential oil did not influence the LPS induced CD80, CD86, and HLA-DR expression (Fig 3).

### Regulation of CD4^+^ T cell polarization and IL-5, IL-10, and INF-γ production with *C*. *obtusa* essential oil

**Fig 4:**
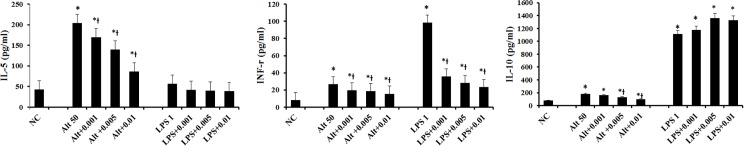
Effects of *C*. *obtusa* essential oil on dendritic cells (DCs) stimulated with *Alternaria* 50 ug/ml (Alt 50) and lipopolysaccharide 1 ug/ml (LPS 1) induced distinctive cytokines from autologous CD4^+^ T cells. *Alternaria*-induced interleukin-5 (IL-5), interferon gamma (INF-γ), and IL-10 production and LPS-induced INF-γ production were significantly inhibited with various concentrations (0.001–0.01%) of *C*. *obtusa*. NT: not treated with essential oil, * p < 0.05 vs. NC, Ɨ: p < 0.05 vs. Alt 50 or LPS 1.

We purified autologous CD4^+^ T cells to coculture them with the DCs. To determine the effects of the essential oil from *C obtusa*, we pretreated the DCs with the oil for 1 hour and stimulated with *Alternaria* or LPS for 24 hours, then we cocultured the DCs with CD4^+^ T cells for 72 hours. *Alternaria* and LPS enhanced the production of IL-5, IL-10, and INF-γ from the CD4^+^ T cells. *Alternaria*-induced cytokine production was significantly inhibited with the *C*. *obtusa* essential oil in a dose-dependent manner. LPS-induced INF-γ production was significantly inhibited with the essential oil, but the oil did not inhibit LPS-induced IL-10 production from CD4^+^ T cells (Fig 4).

## Discussion

Essential oils acquired in nature and contain various components with medical properties such as antibacterial, antiviral, antioxidant and other immunologic effects. [9, 15, 16] Because essential oils are volatile, microencapsulation of essential oils can protect the active compounds against environmental factors such as oxygen, light, and moisture. This microcapsulation also protects the oil from degradation or evaporation and increases their solubility in water. [17] C. obtusa plain oil was about 10 times toxic to respiratory epithelial cells and peripheral blood mononuclear cells than microencapsulated oil. (data not shown) The plain oil compounds might be more volatile and active than microencapsulated one. Stylene maleic anhydride polymer is a synthetic polymer and it has been used as a sealer coat prior to modified release coating and food packing material listed by FDA. [18] It can form a microcapsule by interfacial polycondensation or complex coacervation. *C*. *obtusa* is used for furniture and in cosmetics due to its structural characteristics, natural scent, and anti-inflammatory properties. [12] The essential oil from *C*. *obtusa* contains several monoterpenes that can be promising candidates for new pharmacologic agents. [19] *C*. *obtusa* inhibit the production of nitric oxide and prostaglandin, and the expression of inducible nitric oxide synthase, cyclooxygenase-2, and nuclear factor-kappa B in inflammatory cells. [12, 20] However, the immunologic function of the essential oil of *C*. *obtusa* is not commonly studied. Therefore, in the present study, we analyzed the oil’s effects on Th cell polarization. We demonstrated that the essential oil from *C*. *obtusa* had an anti-inflammatory effect, inhibiting both Th1 and Th2 cytokine production from CD4^+^ T cells. In addition, *C*. *obtusa* inhibited the production of proinflammatory cytokines and costimulatory molecules of DCs.

DCs are one of the key cellular components of immune regulation and determine T cell responses. [1, 21] Chemical mediators secreted from DCs cause differentiation of CD4^+^ T cells into Th1, Th2, or other Th cells, which drives adaptive immune responses. [21] In this study, we attempted to evaluate the effects of *C*. *obtusa* on T cell immune responses. Because DCs are key regulatory cell for T cell polarization, we treated the cells with the essential oil of *C*. *obtusa* rather than directly treating CD4^+^ T cells. *Alternaria*-induced proinflammatory cytokine production and the expression of costimulatory molecules including CD80 and CD86 were significantly suppressed with *C*. *obtusa*.

LPS has been known to activate macrophage and promote of Th1 immune response. [22] LPS strongly induced proinflammatory cytokine production in DCs and Th1 cells, and regulate the polarization of CD4^+^ T cells. *Alternaria*, commonly found and associated with upper and lower airway diseases, activates DCs and induces Th2-type cytokine production. [1, 23] Activation of DCs with *Alternaria* induced Th1, Th2, and Th10 immune responses, particulary of the Th2 cytokine IL-5 (by approximately 5 times compared with negative control). Although we attempted to remove endotoxins from *Alternaria* extracts, the *Alternaria* preparation contained some endotoxin activity (3.7 EU/mg), and the IL-10 and INF-γ production could have been influenced by the endotoxin components in the *Alternaria* preparation. *C*. *obtusa* essential oil suppressed Th1, Th2, and Th10 cytokines in CD4^+^ T cells. Among three cytokines, IL-5 production was most strongly suppressed (IL-5: 75%, INF-γ: 41%, and IL-10: 44%). These results indicate that the essential oil of *C*. *obtusa* can strongly suppress *Alternaria*-induced Th2 immune responses. Inhibition of *Alternaria*-induced IL-6 production from with *C*. *obtusa* may influence the inhibition of Th2 immune response. IL-6 produced from DCs can suppress Th1 polarization and directly induce Th2 differentiation of naïve T cells. [24] However, *C*. *obtusa* did not significantly affect LPS-induced DC activation and LPS-treated DCs enhanced the Th1 cytokine production in CD4^+^ T cells, which means that *C*. *obtusa* essential oil can influence both Th1 and Th2 immune responses. Because we did not evaluate any immunoregulatory properties of *C*. *obtusa*, additional studies are necessary to determine whether down-regulation of IL-6 is sufficient to inhibit Th2 polarization of CD4^+^ T cells or ILS-induced Th1 polarization.

## Conclusion

The principal finding of this study is the Th1 and Th2 immunomodulatory properties of *C*. *obtusa* essential oil. *Alternaria* and LPS can induce production of chemical mediators in monocyte-derived DCs and *C*. *obtusa* influenced both *Alternaria*- and LPS-induced Th1 and Th2 polarization of CD4^+^ T cells. However, only IL-6 and TNF-α production induced by *Alternaria* was inhibited with *C*. *obtusa*. Because the *C*. *obtusa* essential oil influenced both *Alternaria*-induced DC activation and Th2 polarization, *C*. *obtusa* may more strongly inhibit Th2 immune response. These results suggest a novel pharmacological use of *C*. *obtusa* essential oil for treating fungus-induced Th2 inflammatory airway diseases. We need further study to confirm the inhibitory properties of *C*. *obtusa* essential oil in Th2 immune response with other airborne allergens and its possible anti-allergic effects with allergic animal models.
